# Sliding Window Mapping for Omnidirectional RGB-D Sensors

**DOI:** 10.3390/s19235121

**Published:** 2019-11-22

**Authors:** Nicolas Dalmedico, Marco Antônio Simões Teixeira, Higor Barbosa Santos, Rafael de Castro Martins Nogueira, Lúcia Valéria Ramos de Arruda, Flávio Neves, Daniel Rodrigues Pipa, Júlio Endress Ramos, André Schneider de Oliveira

**Affiliations:** 1 Graduate Program in Electrical and Computer Engineering (CPGEI), Universidade Tecnológica Federal do Paraná (UTFPR), Curitiba, PR 80230-901, Brazil; 2 Centro de Pesquisas Leopoldo Américo Miguez de Mello (CENPES), Rio de Janeiro, RJ 21941-915, Brazil

**Keywords:** RGB-D sensor, mapping, omnidirectional, LIDAR, mobile robots, deep-learning

## Abstract

This paper presents an omnidirectional RGB-D (RGB + Distance fusion) sensor prototype using an actuated LIDAR (Light Detection and Ranging) and an RGB camera. Besides the sensor, a novel mapping strategy is developed considering sensor scanning characteristics. The sensor can gather RGB and 3D data from any direction by toppling in 90 degrees a laser scan sensor and rotating it about its central axis. The mapping strategy is based on two environment maps, a local map for instantaneous perception, and a global map for perception memory. The 2D local map represents the surface in front of the robot and may contain RGB data, allowing environment reconstruction and human detection, similar to a sliding window that moves with a robot and stores surface data.

## 1. Introduction

In robotics, visual sensors are responsible for providing robots with environmental data about where they are located. The mobile robot’s skills (such as self-localization and safe navigation through an environment) depend on the quality of its sensors, which support the robot’s perception of obstacles, objects, people, or terrain. In this context, these sensors are often referred to as *sources of perception*. Thus, the mobile autonomy of a robot depends on the amount of information extracted from the environment in which it operates. Path planning tasks, for example, can be better carried out by an autonomous robot if it has several sources of perception at its disposal.

A source of spatial perception allows the robot to identify the distance between itself and the obstacles ahead. Sources of RGB data enable the identification of points of interest in the environment to improve a robot’s odometry, identify people and objects, or even to follow an a prior assigned route. The data fusion from several perception sources can also allow the robot to classify objects for posterior use such as object identification and learning. This approach is discussed in Reference [1], where the authors use RGB sources of perception to build a map linked to a cognitive system, allowing the robot to follow commands such as ’go to the door.’

Cameras are a widely used perception source. However, they present a limited field of view, highly dependent on robot motion to the object or obstacle that must be scanned [2]. The employment of perception sources at the front of the robot is the most common strategy, making the robot vulnerable in dynamic environments or situations that require the robot to move backwards. An alternative solution would be disposing sensors in a rotating platform, independent of the robot’s movement, but this still does not allow the robot to look in more than two directions at the same time. These drawbacks justify the use of an omnidirectional source of perception in dynamic environments, independent of other sources. An omnidirectional sensor is any sensor that can collect data in 360 degrees in 2D or 3D, independently if its readings achieve full coverage or if many sequential readings are necessary. Such a tool allows the robot to identify changes in its environment in any direction.

Mobile robots mainly use sensor data for simultaneous localization and mapping (SLAM) tasks. SLAM consists of building a map of the environment based on data coming from the robot’s spatial perception sources and in identifying the robot’s own position inside this map. The main difficulty in applying the SLAM is due to the cumulative error of odometry that degenerates the perception data. This degeneration is critical in systems that are based on the fusion of perception sources, where the covariance can be wrongly estimated, which leads to inaccurate readings and emphasizes incorrect data. Kalman filters [3] or the Monte Carlo method [4] are techniques that can be applied to minimize odometry errors while building the map. A successful application of a SLAM method still depends on the quantity of available data to map and the distribution of the newly collected data. Therefore, an omnidirectional sensor can also help the optimization of SLAM algorithms by providing data from the whole environment without the use of more accurate sensors.

Accuracy in representing spatial data can also be increased by pairing spatial sensors measurements with RGB data. Images from the environment are used to estimate distance, shapes, objects, and obstacles, which may be used to enable or improve mapping, path planning, or odometry [5]. The environment perception is more reliable with the use of several approaches collecting spatial data (spatial sensors + RGB cameras), which increases the robot’s versatility and compensates the downsides of each source. RGB cameras are light-dependent and falter in poorly lit environments, while spatial sensors are more susceptible to reflective surfaces and light pollution. Gathering data from two or more sensors and using it in a singular representation is called sensor fusion. In this paper, RGB data, represented by images, are gathered with distance data, represented by point clouds, to perform RGB-D fusion (RGB + Distance fusion).

The use of lasers for measuring distances started in the 1970s when LIDAR (Light Detection and Ranging) technology was developed, and it is used since then. This method consists of measuring the phase-shift of an emitted laser beam relative to its reflection on an object (range finder). LIDAR is popular because it provides almost instant distance reading with excellent precision and satisfactory range, as shown in Reference [6]. LIDAR sensor represents 2D data storing each range reading orderly inside a vector. The collected data contains the scanning parameters (i.e., maximum and minimum angles) inside a ’laser scan message’ after a full loop of its rotating mirror (one full scan). The range vector and the scanning parameters contained in this message allow representing each measure as a 3D point with fixed height in a euclidean space. This representation using points is called a ’point cloud.’ Tilting or rotating a laser scan sensor and taking the movement into account when creating the point cloud enables the scanning of fully 3D environments, for example, Reference [7]. Laser scanners are frequently used in mobile robots to perform SLAM, but they are commonly constrained to 2D mapping.

The development of novel methods for omnidirectional sensing usually relies on using an already existing source of perception and its application in a new arrangement, such as using multiple RGB cameras to extract 3D points [5], using mirrors with cameras to achieve omnidirectionality or applying rotation to 3D sensors to increase the field of view [8]. In the recent literature, there are many papers that approach omnidirectional 3D sensors. One of the first published papers [9] used catadioptric panoramic cameras to obtain stereo images of the environment, allowing the identification of which obstacles were near or far from the robot. In Reference [10], we can find a study comparing projections of panoramic acquisitions over point clouds. The authors evaluate the precision of keypoint matching and performance for each method of projection. An omnidirectional 3D sensor is described in Reference [11] using a camera, a projector and two hyperbolic mirrors. Its calibration procedure is also detailed. In Reference [12], an omnidirectional 3D sensor is composed of a hyperbolic mirror, a camera and infrared lasers. The lasers draw a point matrix on the surfaces around the sensor, allowing the cameras to detect the shift in points caused by the distance from the sensor (structured light). In Reference [13], two hyperbolic mirrors, two cameras, and four projectors perform an omnidirectional distance measure, also using structured light from the projectors detected by the cameras to acquire distance data. Another omnidirectional sensor is described in Reference [14], where both stereo cameras and structured light are used to obtain a precise measurement of distance in every direction.

This paper introduces an omnidirectional 3D sensor that takes advantage of RGB and spatial data to perform a novel mapping technique paired with object identification using machine learning, gathering point clouds from a rotating LIDAR and using a camera attached to a hyperbolic mirror. The LIDAR is toppled 90 degrees and fixated over a vertical motor shaft, which rotates, making the sensor omnidirectional. The concept of a local and global map standard in 2D mapping is employed in a new 3D mapping strategy. This strategy, combined with perception sources arrangement, composes the new omnidirectional 3D sensor. The 3D data collected by the LIDAR scanning is represented on a vertical surface in front of the robot, which works as a local map that moves with the robot. The global map is represented by two horizontal surfaces on the ground and at the ceiling, using the data represented on the local map to create a full map of the environment. Object identification applied to the panoramic images is used as a demonstration of how RGB data can be used to improve 3D mapping. This data can also be used to represent recognizable objects on the map and identifying dynamic entities (people) to assign obstacles better when mapping.

## 2. Problem Statement

Many researchers have developed different sources of perception for 3D mapping. In Reference [15], a 3D mapping algorithm is designed to generate surfaces using point clouds and RGB data. The Octomap is described by Reference [16], which is a mapping strategy that aims at building 3D maps quickly and dynamically based on any spatial sensor. The Octomap is a tridimensional grid in which each cube (called voxel) is classified as occupied or empty based on the presence of points from point clouds. A fusion algorithm of RGB-D and odometry data presented by Reference [17] uses a hand-held Microsoft Kinect for the reconstruction of indoor environments. The work described in Reference [18] is an RGB-D sensor using stereoscopic RGB cameras. The cameras rotate in 10 revolutions per second, allowing a full panoramic view, but only in grayscale. However, the mapping techniques are not adapted to explore the benefits of omnidirectional perception, constraining the robot mobility, and degenerating the perceptions precision.

Several papers develop non-traditional mapping techniques adapted to different environments. In Reference [19], a visual localization algorithm for environments without many landmarks is designed to build a surface map supporting path planning for a six-legged robot over rough terrain. In Reference [20], a geographical laser scanner to map the interior of rooms is used for fusing acquired 3D data with RGB and temperature data. The authors use voxels as representations to build the map. Full room scanning takes up to a minute, with the robot standing still. A coordination strategy for multiple robots along with a novel 3D mapping technique was developed in Reference [21], by using the contour of walls and objects to define planes in 3D space. The surfaces are identified from point clouds collected by 3D cameras fixed on the robots. In Reference [22], a mapping method is described using previous knowledge of the environment. Again, representations are limited to mix perception with known maps to achieve a more concise initial planning, where benefits of omnidirectional perception are disregarded.

The coupling of visual and depth perceptions is discussed in RGB-D SLAM, where many approaches are presented to achieve a more detailed environment understanding. For example, in Reference [23], the authors try to develop a practical method for RGB-D SLAM with a Microsoft Kinect 1.0 using only visual or spacial methods, concluding that to reach a precision similar to more expensive sensors and exhaustive optimization is required. Another work involving the Kinect can be found in Reference [24], where the author uses a process of RGB-D SLAM with the help of a GPU to achieve 22 Hz of acquisition registering frequency. A novel method of monocular SLAM with relocalization (loop closure), claiming real-time processing for the trajectory calculation, is presented in Reference [25]. An RGB-D SLAM for aerial vehicles with embedded processing in real-time is developed in Reference [26]. In Reference [27], the authors develop an RGB-D SLAM for a hand-held sensor, using points and planes as primitives to build the map. Finally, an Asus Xtion (RGB-D) using the events reported by a dynamic vision sensor (DVS) to focus the 3D processing captured by the camera is presented in Reference [28]. Thus, the concept of fusion between visual and depth perceptions is very relevant to improve robot interaction. However, previous approaches are focused only on coloring environment representations without enhancing understanding.

More detailed environment maps allow the introduction of complex interactions, for example, detecting future collisions [29] or pushing objects [30]. In this context, environmental characteristics are crucial to achieving intelligent behaviors. There are several techniques for identifying objects in RGB images and deep learning techniques [31], such as convolutional neural networks (CNN) [32], are widely used. These techniques allow you to answer simple questions when processing an image, such as whether there is a cat or not in an image, or to detect objects relevant to the robot, such as doors [33]. So that this information can be used to execute some action, such as opening them, the object detection and movement prediction can exist even on basic two-dimensional maps [34], though with limited capability.

This paper aims to present a novel mapping approach based on a sliding window to represent the environment through the inputs of an omnidirectional RGB-D sensor. The proposed mapping strategy allows the use of machine learning classification and the introduction of intelligent behaviors in mobile robot navigation.

## 3. The Proposed Omnidirectional RGB-D Sensor

**Figure 1:**
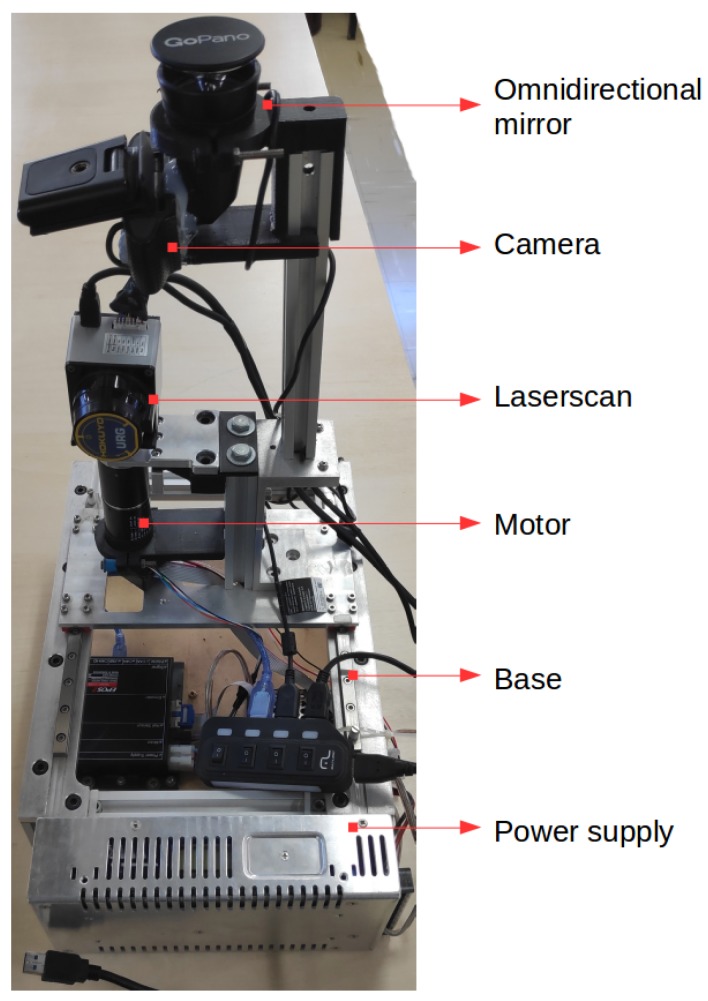
Layout of omnidirectional RGB-D sensor.

The sensor’s layout (architecture and hardware) is a prototype, where no efforts to optimize electrical and mechanical design have been taken into account. This paper aims to develop and validate the interaction between actuated LIDAR and RGB through data fusion algorithms. The sensor is mounted on an aluminum profile structure (box) as a base, which holds processing and power components inside and sensors and actuators on top. This sensor prototype can be seen in Figure 1.

The RGB camera used in this paper is a Logitech C920 HD camera, fixed in front of a hyperbolic mirror to enable full panoramic images. These were set at the end of a shaft on top of a aluminum profile structure. The motor and the laser scan sensor are also on top of this box. The engine is vertically disposed, with its shaft spinning the sensor fixed at its end. The sensor is toppled on top of the motor shaft doing vertical readings. Three-dimensional printed supports are used to couple the LIDAR sensor on the motor shaft, the RGB camera and the hyperbolic mirror on the aluminum profile shaft and the motor base on the top of the box. With the motor movement, sequential readings can be carried out, covering every direction and allowing an omnidirectional acquisition. The motor used is a Maxon EC32 brushless servo motor controlled by an EPOS2 24/5 digital positioning controller. The laser scan sensor used is a Hokuyo URG-04LX LIDAR. The base of the box holds the EPOS2 controller and power supplies.

The two primary sources of perception and the actuator provide omnidirectional 3D and RGB data. The laser scan gathers a column of 511 3D points at a specific angle. The motor spins the Hokuyo, sweeping the environment, allowing the full or partial scan of the environment. The 3D data is the input for a robot mapping strategy developed for slow sweep scans of a region of interest, defined as a 3D vertical window in front of the robot, which allows the creation of a 3D surface representing the space in front of the robot. The RGB panoramic image is input for an image detection algorithm (Darknet YOLO [35]) that finds objects and people on the image and informs the region of identified objects to the surface map. This information is also used to coherently clear registered data from moving objects, diminishing map pollution. This functionality is an additional feature offered by the mapping strategy and sensor combo.

### 3.1. Planar Perception

**Figure 2:**
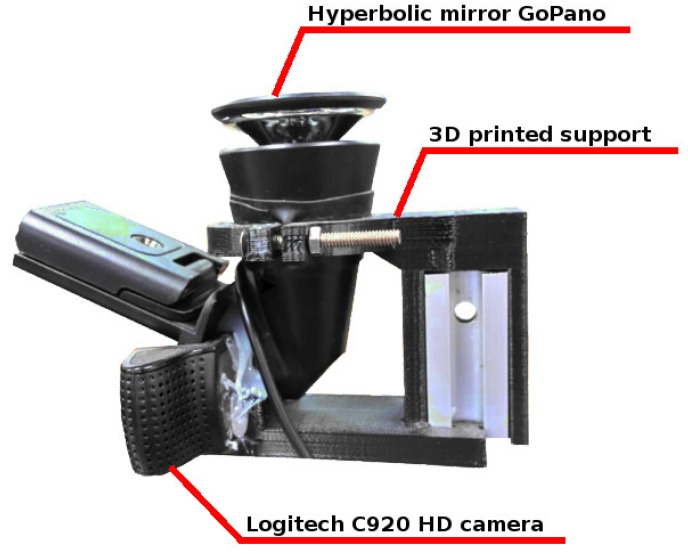
Part of the proposed sensor for capturing 360° RGB images.

The RGB sensor was chosen due to its resolution, which can reach 1080p at 30fps (frames per second) with a field of view of 78 degrees. These characteristics make this equipment ideal to be added to a hyperbolic mirror, allowing the generation of an image with a 360° field of view. The camera was attached to the hyperbolic mirror using a 3D printed support designed for this project. A picture of the whole set can be seen in Figure 2.

**Figure 3:**
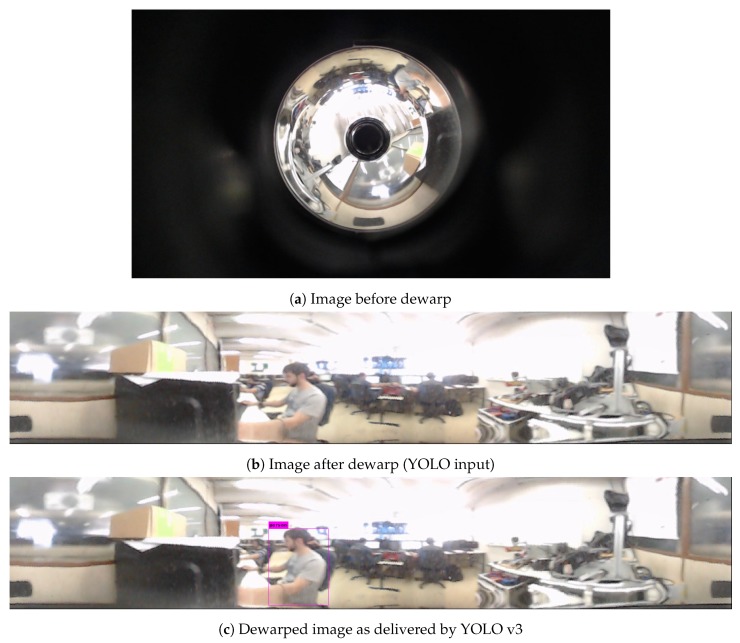
Conversion of the camera acquisition to panoramic image, which is fed to the YOLO object detection.

This set is responsible for acquiring the images in 360 degrees. Such images will be used by the object detection algorithm to identify people in the environment. Figure 3a presents an image as captured by the camera. As can be seen in this figure, the image presents regions that do not concern the environment (black region). Thus, they cannot be used by YOLO object detection because this algorithm was not trained to identify objects when they are upside-down or distorted in the image. A method was developed to extract and convert the omnidirectional image to a linear image, circumventing this problem. This operation is known as *dewarp* [36,37].

The algorithm developed to perform dewarping has as its input an image from the camera looking through the hyperbolic mirror, with a resolution of 1920 × 1080 pixels as in Figure 3a. The output of the algorithm is an ultra-wide image, where all objects in the image lie at the same angle (Figure 3b). The algorithm takes the input image and finds the center of the warped circle that represents the image through the mirror. The outer and inner radius is computed; anything below the inner radius is discarded. The circle is divided into many arcs that are iterated and reshaped into rectangles. Every pixel of each arc is mapped to the resulting rectangle, forming a stretched but upright image.

After processing the image provided by the sensor (Figure 3a), the dewarped image feeds it to the classifier YOLO [35]. The classification employed was done as an example of what fusing RGB and 3D data allows, and any computer vision algorithm using the dewarped image to extract information can be applied to improve mapping or navigation. The YOLO classifier is a convolutional neural network with multiple layers, aiming to identify objects in an RGB image. YOLO is a deep learning algorithm trained with the COCO database [38]. YOLO can identify objects and humans over the dewarped representation (the image processed by the dewarping algorithm and shown in Figure 3b). As a result, YOLO object detection returns the position of identified humans and objects, as can be seen in Figure 3c. In this figure, the known location of a person is marked in pink. The bounding boxes of objects detected by YOLO are input to the developed mapping strategy described in Section 4.

### 3.2. Spatial Perception

**Figure 4:**
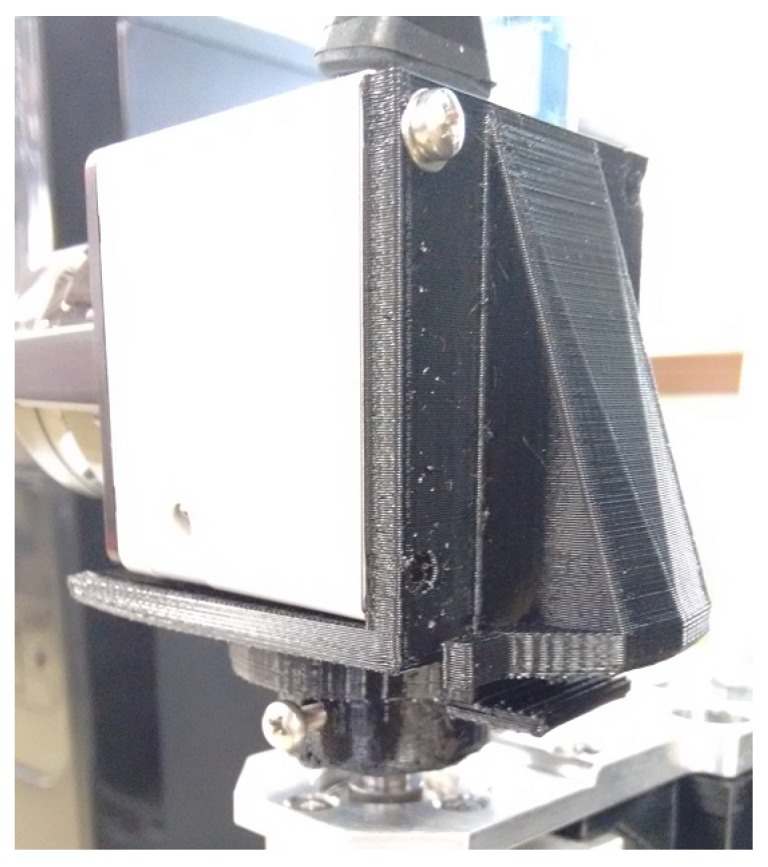
3D printed support for fixing the Hokuyo LIDAR toppled on top of the motor shaft.

The Hokuyo URG-04LX LIDAR laser scan sensor is responsible for gathering distance measurements along a horizontal arc in front of itself. The LIDAR (Light Detection and Ranging) is a sensor with only one range sensor (infrared laser) vertically positioned, which is queried while a 45° oriented mirror spins over it, reflecting the light where the mirror is pointing and allowing coverage in a 240° arc. Each revolution of the mirror takes 1/10th of a second and gathers 511 points, composing a laser scan acquisition. Commonly, it is used in robotics to implement 2D mapping for flat environments with fast self-localization. On this prototype, a different approach is taken: the sensor is toppled 90° collecting ranges in a vertical arc and it is then rotated by a motor to sweep the environment (actuated LIDAR). The Hokuyo is fixed over the shaft of the motor using a 3D printed support (shown in Figure 4), and spin around its center of mass.

The Maxon Motor EC32 brushless motor was set to velocity profile, controlled by an EPOS2 24/5 controller. The rotation performed is done in a back and forth fashion as not to entangle the wiring. Rotation limits are defined by the mapping algorithm, being the size of the local mapping window. The motor’s angular velocity can be configured via parameters. Power supplies are fixed at the base of the prototype and provide power to the Hokuyo and the EPOS2 controller.

The interaction between the motor’s rotation and the Hokuyo allows the slow sweeping over the environment. The Hokuyo collects a column of range measurements while the motor spins in a constant velocity, covering the whole environment or a section of it. The angle of the engine, along with the collected ranges, allows the representation of the data as a 3D point cloud, which is used as the 3D input for the mapping strategy.

## 4. The Proposed Strategy of Sliding Window Mapping

Because of the innovative aspect of the proposed mapping strategy, it is hard to compare against other well-established 3D mapping techniques, since there are features that are exclusive to each approach. The contribution of this mapping strategy is to allow actuated LIDAR sensors to match 3D cameras, offering 3D mapping without making use of point cloud stitching, and taking advantage of underused aspects of actuated LIDAR, such as their flexibility in regards of field of view. The method of map building proposed in this paper allows a low-memory spatial representation limited by the size of the instanced grids. It is still able to perform image processing that can be immediately translated into 3D, as the local map holds a section of the panoramic image. The proposed mapping strategy also has the most used structure of 2D map representation (the occupancy grid) as a direct output, significantly spreading the range of tools and packages available to be used along with it.

**Figure 5:**
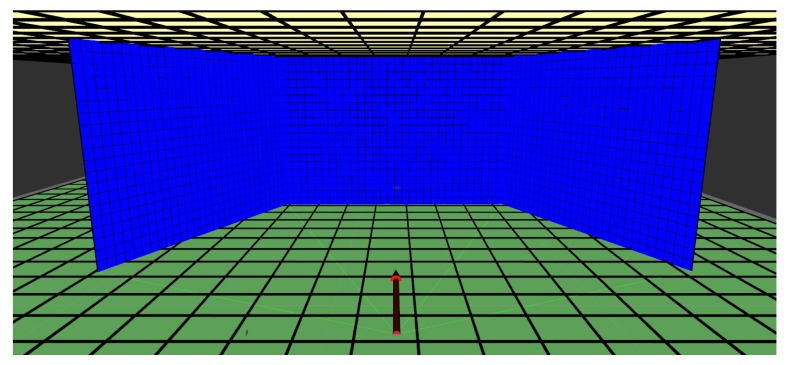
Empty grids for the local map (blue) and global map (green for ground, light yellow for ceiling) of the proposed mapping strategy.

When using an actuated LIDAR to map the environment, the most common way is to point it forward and tilt the LIDAR laterally, rolling the laser scan sensor on its side back and forth, or horizontally, tilting it up and down continually. Common mapping strategies employs the LIDAR motion in this fashion, performing a full period of movement while gathering points, fusing them all on a point cloud after one cycle is complete and then use mapping strategies oriented for point cloud sensors, which are plenty. This proposed mapping algorithm focus on LIDAR toppled on its side sweeping vertically, from side to side, and does not update once every cycle, instead assigning data every scan. Representing data on grids allows memory usage to be constant during mapping because the grids do not change size during execution. The represented data is transformed as the robot moves to compensate movement and keep detected obstacles coherent with the environment. Figure 5 shows the empty maps of the proposed mapping strategy.

**Figure 6:**
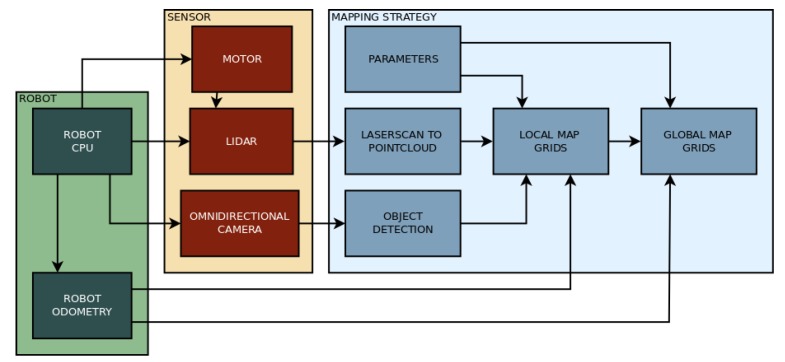
Flow chart of the proposed mapping strategy.

The proposed 3D mapping algorithm is developed for mobile robots with actuated LIDAR perception, and it uses RGB data to identify humans. The mapping strategy consists in reconstructing the environment in front of the robot with a vertical surface (similar to a local map in a 2D mapping strategy). This vertical surface is created by three grids forming a ’sliding window.’ These grids move together with the robot and update the represented obstacles. The sliding window is composed of a central grid and two adjacent and angled grids. The data from this surface are used to assign obstacles in a global map, which can also be converted to an occupancy map. This global map is composed of two horizontal grids, one representing the ground and obstacles that the mobile robot needs to avoid, and the other corresponding to the ceiling, which will not affect navigation. This later grid is only used to assure environment reconstruction. The vertical surface in which the environment in front of the robot is represented is called ’local map,’ and the horizontal surfaces to reconstruct the whole environment is called the ’global map.’ The global map is then used for deriving the occupancy map. The flow chart on Figure 6 shows how the mapping strategy relates to the sensor and the robot.

The map grids are defined by the ’Grid Map’ ROS package [39]. The grid object can have many layers, and each layer is defined by a matrix having the size of the grid. One or two grid layers can be visualized on ’rviz’ by defining an elevation layer and an optional color layer. Rviz is a data visualization software distributed with the ROS framework. ROS stands for Robot Operating System [40], and it is the main framework over which the mapping strategy operates.

The mapping strategy follows many steps from gathering the data until representing it on the maps. First, a transformation tree (tf tree) is defined using data from the motor’s rotation and sensor position, establishing a ’sensor’ reference and its relation with ’base link,’ which is the most commonly used base reference for mobile robots, usually defined in its center of mass or central axis of rotation. The references for each grid and the global ’map’ reference are also created using user-defined parameters. The transformation tree is used to support conversions of spacial points in the mapping strategy. The transformation between the ’odom’ frame (the robot’s initial position) and the ’base link’ is carried out by the package handling the robot’s odometry.

**Figure 7:**
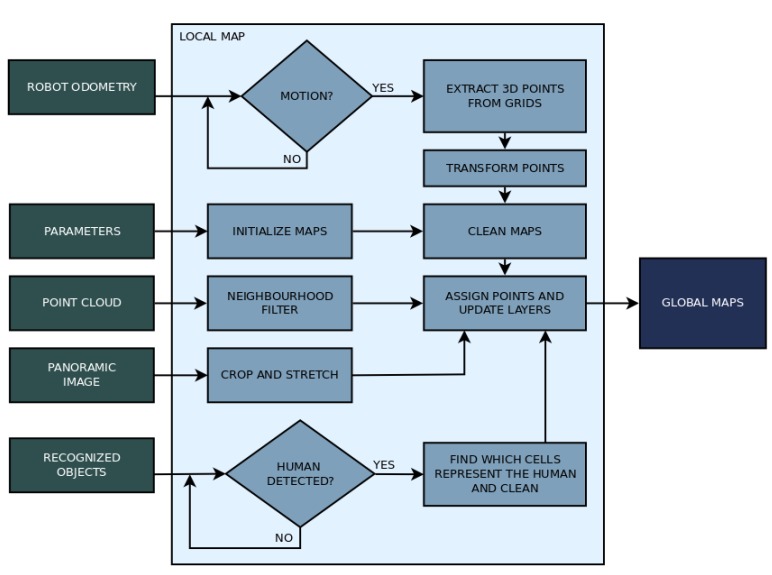
Flow chart of the processes involved in building the local map.

The local map has five significant steps to its operation. It starts by loading parameters as well as initializing every grid and essential variables. Then, it enters the main loop, which checks for incoming data: point cloud data, image data, odometry data, and identification data, applying the corresponding action depending on what it receives. The point cloud data is filtered and processed to define the shape of the grids. The RGB panoramic image data is used to update the grid’s color, matching the image with the grids’ location. The odometry data tells the mapping algorithm if the robot moved. If any movement was detected, a procedure compensates the movement by transforming the grids to keep obstacles fixed (about the global map) while the base of the grid where they are represented moves. The identification data arrives as a list of objects identified by the YOLO algorithm, which is iterated to store the four sides of the bounding boxes that represent humans, which are then used to clean the respective section of the grids. The Algorithm 1 describe the local mapping strategy. The flow chart in Figure 7 shows how the main steps of building the local map.

The laser scan data collected by the Hokuyo sensor is transformed to point cloud data considering the robot’s center of mass (base link). This conversion is done every scan, without waiting for two or more readings as usually done when mapping with actuated LIDARs. Therefore, as scan readings are represented in the same 3D space as the robot, the data can be directly assigned to the sliding window (local map). Before the assignment, though, a fusion operation (the mean of neighbor points) is applied to reduce the number of points to be iterated, with a radius corresponding to the local grids’ cell size, so that every scanned cell receives one resulting point. The assignment is mathematically computed using the grid’s spacial configuration. The three grids of the local map represent the points as elevation, creating a surface that represents the obstacles in front of the robot.

The robot’s position is monitored so that the obstacle position is compensated when the robot and the local map move. This compensation is done by a transformation on the elevation data represented on the grids. Non-empty cells are converted back to 3D points, transformed, and then reassigned. As a consequence of this, the obstacles stand still relative to the ground, even though the grids are moving with the robot. The RGB data read by the camera attached to the hyperbolic mirror is received and dewarped. Thus, a corresponding section of this image is assigned to the three grids of the local map, assuming one pixel for one grid cell, meaning the image needs to be resized before attaching it to the local map.

The classification is done by YOLO neural network classifier over the dewarped RGB image with full resolution. The neural network was trained to identify people so that the mapping strategy may use this data to clean the map and avoid dynamic entities. As explained above, every frame processed by the classifier generates bounding boxes that define the region where a person was detected. These bounding boxes are the input for the mapping strategy to erase the data read as not to compromise environment reconstruction and path planning. The bounding boxes are also represented within a ’debug’ layer of the local maps, as additional info in the case of a path planning algorithm is employed. The bounding boxes that represent humans and objects from the YOLO ROS package are received, their corresponding locations on the map are calculated, and the correct operation is applied over the local map.

**Algorithm 1:** Local sliding window of the RGB-D mapping strategy.

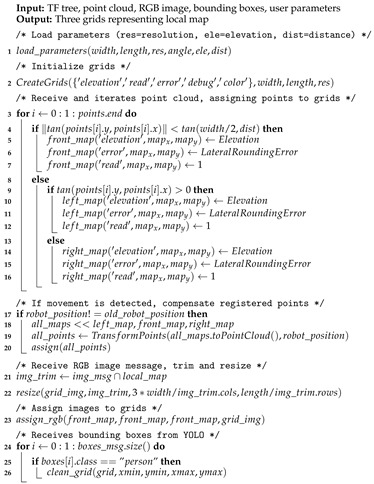



The local map is used to assign obstacles to the global map, which will iterate on non-empty cells and assign the correct elevation on one of the two global grids, floor and ceiling, based on elevation from the ground. The global floor grid is also converted to an occupancy map to assist navigation.

The global map has as inputs the user parameters, local map grids, and robot odometry. It initializes its two base grids, one for the floor and other for the ceiling, based on the configuration parameters. The two grids are horizontal to the ground, apart from each other by a defined setting and can reach an elevation only enough to touch each other based on a threshold parameter, which will determine which obstacles are blocking the movement of the robot and which will allow the robot to navigate under them.

**Figure 8:**
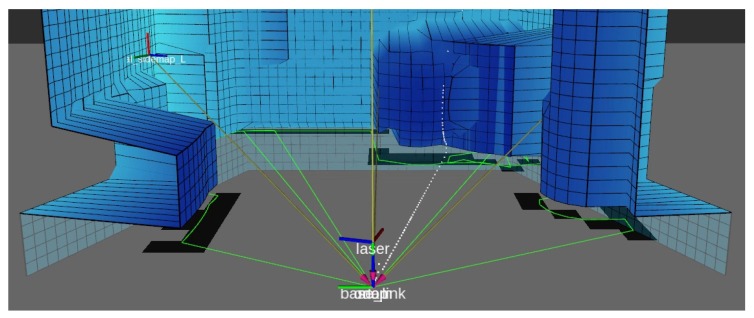
The green lines are the polygons that define the robot’s sight. Each square of the global map which has its center inside any of the three polygons will be cleaned. The local map was shifted one square up so that it is possible to see that the polygons follow the contour of the local map.

The global map monitors the three grid messages from the local map and the robot’s odometry. When a new grid arrives it is iterated over assigned cells (ignoring empty cells) and the represented obstacles are attached to the respective map based on height (ground or ceiling). Using the non-empty cells that are assigned on the local map, a robot ’sight’ is simulated, creating three polygons with sides as each occupied cell and the center of the robot. Cells from the ground grid that are inside these polygons are cleaned to represent no obstacles detected in its sight. Images of the sight polygons can be seen in Figure 8.

**Figure 9:**
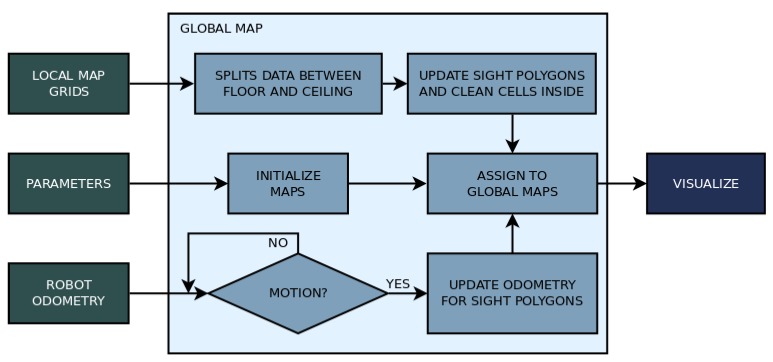
Flow chart of the algorithm that builds the global map.

If the robot moves, the global map only updates the robot center and yaw variables so they can be accounted for on the sight procedure and obstacle assignment. The flow chart in Figure 9 shows the essential steps to build the global map.

The occupancy map is generated using a procedure that belongs to the ’Grid Map’ package, which uses a defined grid as input and produces a corresponding occupancy map. Small differences of elevation can be represented with a slightly higher cost on the occupancy map, or a threshold elevation can be used to determine occupied cells.

**Figure 10:**
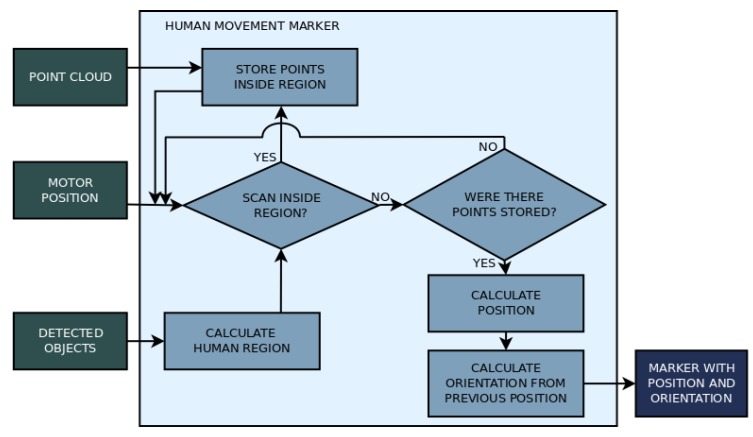
Flow chart of the human movement prediction algorithm.

Dynamic environments offer two main difficulties to mobile robots: it is difficult to maintain coherence while mapping due to moving objects, and it is also hard to navigate without bumping into obstacles or humans. Because of this, a human movement prediction algorithm was also implemented as example of application for the human recognition process, set apart from the mapping algorithm, to verify the possibility of using the identified humans in the image to improve path planning. This algorithm gathers the center of humans identified by YOLO using the bounding boxes data and laser scan data in two sequential acquisitions to infer the direction of movement. An arrow marker is used to represent the position and direction on the map. The flow chart in Figure 10 explains the algorithm to find human position and orientation.

## 5. Experimental Evaluation

The proposed mapping strategy is composed of many complex operations. From representing an obstacle on the map to keep it cohesive while moving, many aspects need experimental validation so that its functionality can be displayed.

Five different experiments were envisioned to validate the mapping algorithm. The first corresponds to an environment reconstruction and its primary goal is to prove the local map capability of representing the robot’s surroundings. The second experiment is to create a map while moving to validate the movement compensation. The third experiment confirms the human detection algorithm, showing the map when a human is present and cleaning the map where a human is identified. The goal is to verify if the human has not interfered in the reconstruction and is correctly represented on the ’debug’ layer, so that path planning algorithms are still able to avoid the human even though he/she is not shown on the elevation map. The fourth experiment evaluates if a human movement is correctly represented on the map by the movement marker and validates the markers as support for path planning in the presence of dynamic objects. The last experiment assesses the accuracy of the represented data in quantitative analysis.

**Figure 11:**
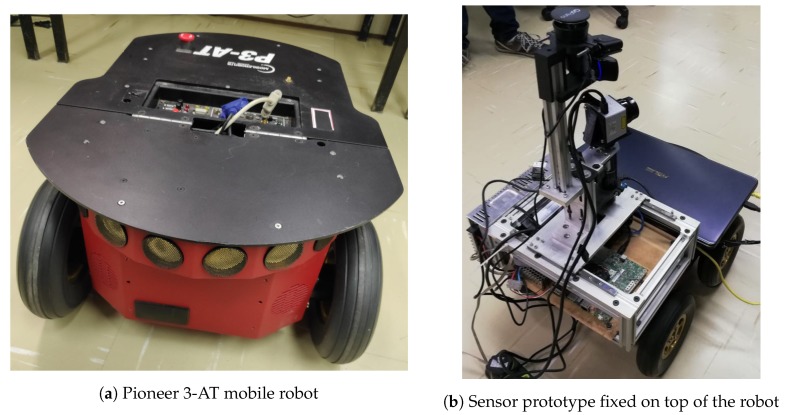
Experimentation platform.

The experiments were carried out with the proposed sensor prototype fixed on top of a Pioneer 3-AT (P3AT) robot (Figure 11a). Both the P3AT robot and the sensor were connected to a notebook running ROS also placed on the top of the robot (Figure 11b). The laptop was connected via wifi to a desktop in the same network, also running ROS, allowing the visualization of ROS topics on rviz. Footage of similar experiments can be found in a YouTube video available as supplementary material to this paper at https://youtu.be/lD2Ona6xw4s.

### 5.1. Reconstruction Experiment

**Figure 12:**
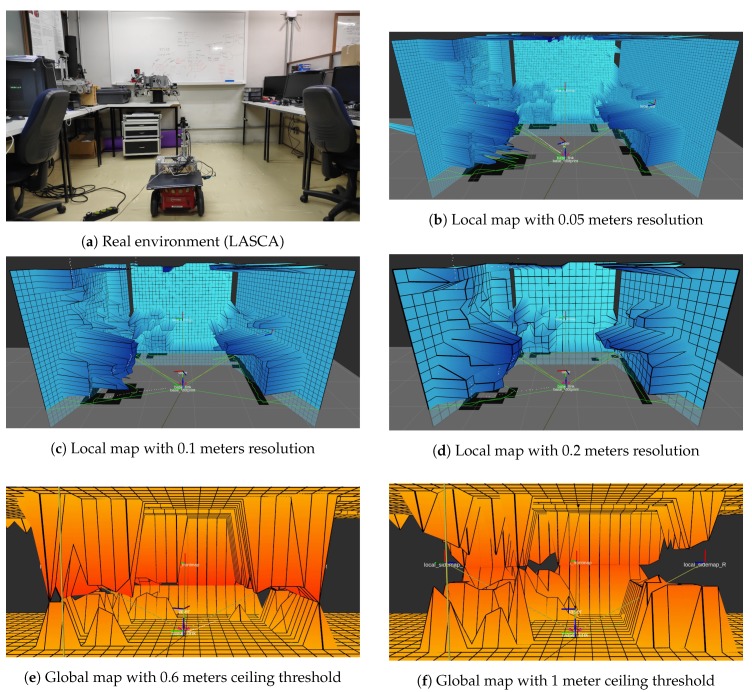
Results of the reconstruction experiment without using RGB data.

The first experiment aims the validation of the mapping algorithm and the 3D representation of the environment. The robot carrying the sensor was put in front of a cluttered environment (LASCA lab, which can be seen in Figure 12a) so that the sweeping LIDAR could take 3D acquisitions. Data from the RGB camera was not used in this experiment since the goal is to verify how close the generated surface resembles the actual environment. Since the mapping strategy has several configuration parameters, some of them were varied to show their use. The grid resolution parameter for the local map was varied between 0.05, 0.1, and 0.2 m for the local map and had a fixed value of 0.2 m for the global map, to assess its impact over the resulting grids. The ceiling threshold parameter for the global map was also varied between 0.6 and 1.0 m to show its impact on the resulting navigable area. Figure 12b–d show the results.

As can be seen in Figure 12b–d, which correspond to the resulting local map generated by a single scan sweep, the sensor along with the mapping strategy is able to reconstruct the environment shown in Figure 12a, resembling the real disposition of obstacles quite reliably and representing them on the respective grid. The different values of resolution impact the overall level of detail though the broad shape of the environment is consistent between all three experiments. The two generated global map grids, shown in Figure 12e,f, also represent the same environment, now ’split’ between ground and ceiling. When the ceiling threshold parameter of the global map was set to 0.6 m, the robot understands the area under the tables (right side of the figure) as navigable. This is not the case when the ceiling threshold parameter is set as 1 meter, which is higher than the top of the tables, making the space under them being marked as occupied.

**Figure 13:**
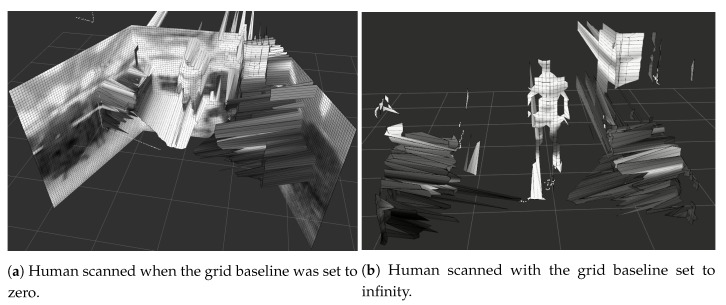
Impact of the ’grid baseline’ parameter.

Another parameter that may be used for reconstruction is the grid baseline, which can assume zero or infinity values, changing how the grid will be visualized, though it does not influence the results. Figure 13a,b shows the difference of the two options for grid baseline.

Figure 13a,b shown the grid baseline parameter will impact the visualization of the grids of the local map. It’s only a visual option. For calculation of regions of interests, sight, and assignments on global maps, this parameter makes no difference.

### 5.2. Navigation Experiment

**Figure 14:**
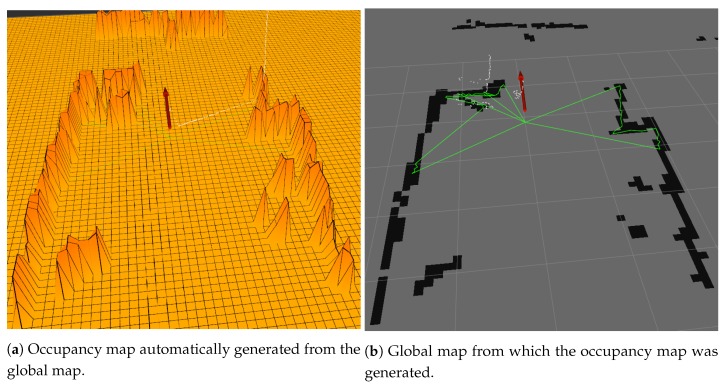
Occupancy grid map from global map.

The global maps allow navigation over the environment by being converted to occupancy grid maps in real-time by the ’Grid Map’ package, in which the mapping strategy is heavily dependent. Figure 14a shows the occupancy map of the converted global map of Figure 14b. The occupancy map allows for most SLAM tools and path planning algorithms to be used with the proposed mapping strategy. Parameters like ’maximum elevation step’ can be configured so that the robot understands ramps as navigable or to filter out small bumps in the grids from the occupancy map.

**Figure 15:**
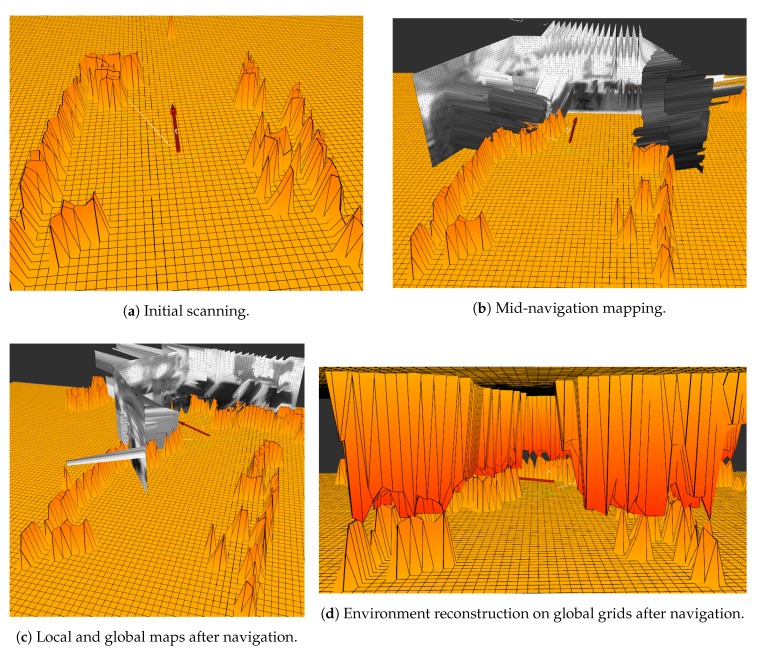
Results of the navigation experiment.

Navigation experiments were done to show the capability of the sensor and mapping algorithm to represent indoor environments. The navigation experiments were carried out by moving the robot through the room and making a 90 degree turn in the end to assess obstacle reliability when turning the robot away from the already scanned environment. Figure 15 shows the resulting global map experiments done with the robot moving through the room while scanning the environment.

As Figure 15 shows, the mapping algorithm can successfully build a global map of the environment while navigating. As the experiment was done in a very cluttered environment — a room with many chairs, desks and other objects on the ground — the resulting map only grossly resembles the environment.

### 5.3. Human Detection Experiment

**Figure 16:**
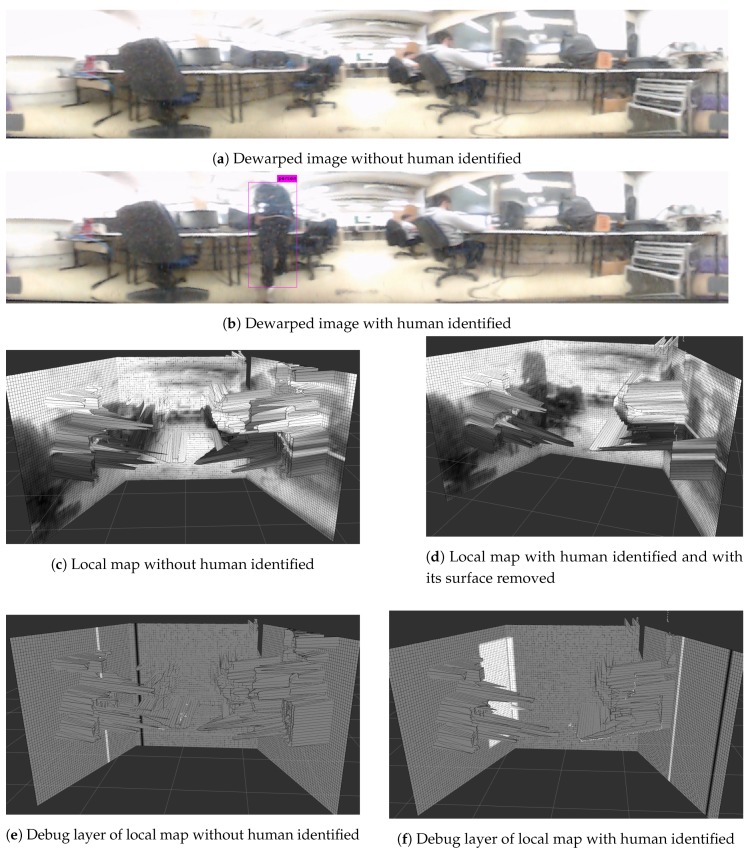
Results of the human detection experiment showing environment before and after a human is identified. The surface generated from its scanning is removed from the local map.

Human detection by the mapping algorithm was done so that dynamic entities could be erased and would not generate an obstacle represented in the local and global map, thus leading to map pollution. The same experiment as reconstruction was performed, though this time a human was present in the scene, to validate human detection. Figure 16 shows the resulting local map, the dewarped image from the camera, and the human shown in the ’debug’ layer. Humans who were seated were not detected by the YOLO algorithm.

As Figure 16 shows, the human was successfully removed from the map, generating no obstacle pollution. The debug layer still accuses the human presence, allowing the global map to mark the space occupied by him as not navigable. The two black and white columns are shown in the debug layer are the cleaning cursor (white) and sensor rotation cursor (black) and follow the sensor’s motion. The YOLOv3 has an accuracy of 28 mAP (mean Average Precision) [35], meaning the algorithm can skip some frames where a human was present but was not identified.

### 5.4. Movement Prediction Experiment

**Figure 17:**
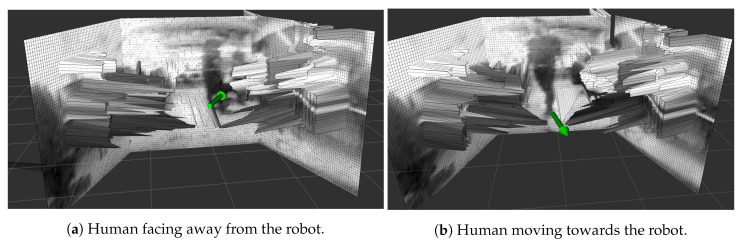
Human movement experiment. The green arrow shows position of the human erased from the map and points to the predicted direction of movement.

An experiment was carried out with a moving human in front of the robot while the sensor is scanning, validating the last feature of the proposed mapping algorithm and sensor, the human movement marker. The movement is inferred by combining the human detection and range data from the laser scan sensor. The sensor sequentially collects several points when scanning inside a human bounding box obtained from the human detection algorithm. Figure 17 shows the results when a person moves in front of the sensor while the robot was mapping the environment.

The figure illustrates the marker (in green) obtained from movement direction data where the rotating sensor last saw the human. Two sequential readings allow the calculation of velocity and direction of movement, essential data for path planning algorithms that support dynamic entities.

## 6. Accuracy and Precision

An experiment was envisioned to evaluate the representation error of the proposed mapping strategy. Because of the discrete nature of the mapping strategy, a maximum error of at least half the size of a grid cell is expected when assigning obstacles (half-cell error). Thus, this quantitative analysis is focused on the only continuous dimension: the elevation assigned to every grid of the local map. The elevation represented on the grids of the global map is also discrete, despite being able to assume continuous values, because it is derived directly from the line of the local grid representing the obstacle to be assigned.

The experiment consists in measuring the distance of a box represented in the map and compare it with its actual range, using different positions to cover every part of the local map. The box used for the experiment is roughly 40 cm × 40 cm in size and was put on top of a 10 cm pedestal.

**Figure 18:**
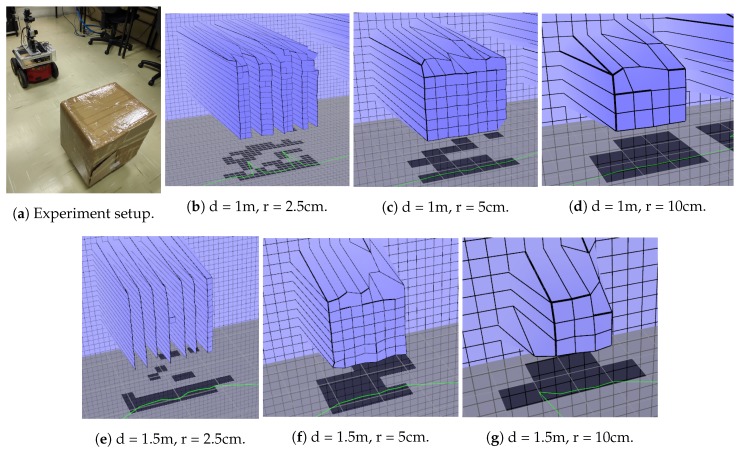
Box distance measurement experiment showing the experiment setup (Figure 18a), scans of the box with different values of distance (d = 1 m and 1.5 m) and local map resolution (r = 2.5 cm, 5 cm and 10 cm). The black squares represent the box on the global map. The green line represents the sight polygon.

First, a distance error analysis was done based on the position of the box. The box was positioned a fixed distance away from the center of the robot and scanned three times. Cells representing the box were measured, allowing the calculation of mean and standard deviation. Three different values for local and global map resolution were tested, as well as two different distances between box and robot. Table 1 shows the results. In Figure 18, images of the experiment can be seen.

By analyzing the values in Table 1, it can be noticed that there is a systematic error present in the measurements. One of the reasons is the box being positioned below the sensor. The sensor on top of the robot stands 57 cm tall while the box on top of the pedestal stands 50 cm tall. This means that the sensor also captures points on top of the box, resulting in the top row of the box on the map to be represented a bit further away. This effect can be seen on the scans of the box in Figure 18. This is an inherent characteristic of the mapping strategy.

**Figure 19:**
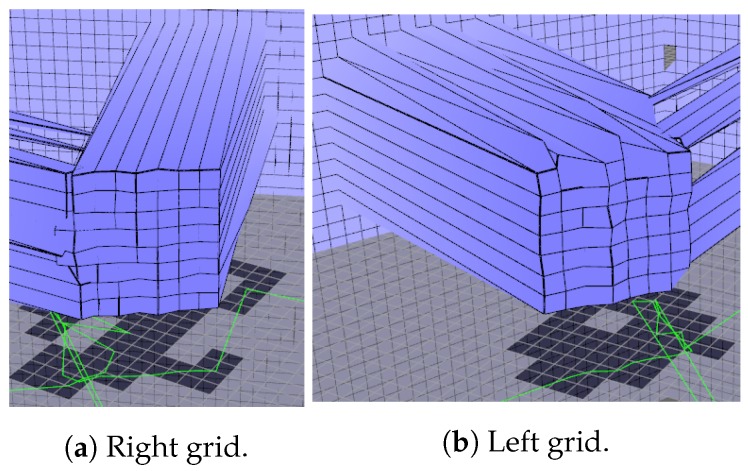
The box represented on the local map when seen by the right (**a**) and left (**b**) grid.

A similar experiment was done to verify if the representation is consistent for the left and right grids of the local map. Scans of the box were done after turning the robot 45 degrees left and right so that the box be represented in the lateral grids. Local map resolution was set to 5 cm. Lateral grids were disposed of with a 75-degree angle from the front grid. Results are presented in Table 2. The box represented in the lateral grids can be seen in Figure 19.

The results in Table 2 show that turning the robot away from the box impacts the precision of the readings slightly, but introduced errors are minimal in magnitude when compared with sensor error and the inherent half-cell error due to the grid.

Overall, several steps of the mapping strategy introduce errors to the represented map. The primary error sources are the LIDAR measurement error, the motor encoder error, the half-cell error in local and global grids, and the odometry error from the robot using the sensor. All of these error sources affected the results of the experiments done in this section. The random error found in the tests seems to be a bit bigger than expected when comparing the Hokuyo URG-04LX maximum random error of 3 cm (for distances around 1 m) found in [6,41,42] to the 7 cm standard deviation present in the results. Still, it seems adequate when considering the sensor is toppled and there could be errors when positioning the box.

## 7. Conclusions

This paper has proposed an omnidirectional sensor composed of an actuated LIDAR and a camera, as well as a mapping strategy using 3D and RGB data using grid representation. The data from both perception sources are merged in the represented grid, supporting a novel mapping strategy of a surface in front of the robot. The surface was able to successfully represent the environment, allowing 3D mapping during navigation for mobile robots. Though there is much work to be done, the current results are already promising. The proposed mapping strategy was based on the described sensor, taking advantage of its sweeping scanning style and omnidirectional fashion. Several operations, such as human removal and movement prediction, were done to show the possibilities of this sensor and mapping strategy combo.

For future work, a more robust dynamic object movement prediction can be made, transferring the acquired data to the global map so that the mobile robot can make decisions when planning a path. Object detection can also be done so that the algorithm can move towards semantic mapping, representing objects such as doors and chairs on its environment.

The sensor prototype can be vastly improved, as it was made only for research purposes with no attention to size, weight, or electrical project. A compact version of the sensor, together with a GPU development board like the Nvidia Jetson Nano, for example, should be able to perform the same functionalities with much less power consumption while being more compact. Future work with GPGPU (General Purpose GPU) processing can also be employed for the mapping strategy in operations such as sensor fusion and image feature extraction, as shown in Reference [43].

Overall, actuated LIDARs still feature numerous research opportunities and contributions to mobile robots. The potential of these devices is far from being fully explored and it seems that every day so many new doors are opened with advances in parallel processing and deep learning that it is not possible to explore all the possibilities. 

## Figures and Tables

**Table 1:** Box distance shown in map and local map resolution experiment results. Experiment done with box distance 1 m and 1.5 m. Results in meters.

Local Map Resolution	Mean		Std. Dev.		Error	
	1 m	1.5 m	1 m	1.5 m	1 m	1.5 m
2.5 cm	1.0204	1.5238	0.0395	0.0112	0.0204	0.0238
2.5 cm	1.0236	1.5316	0.0472	0.0508	0.0236	0.0316
2.5 cm	1.0219	1.5321	0.0532	0.0451	0.0219	0.0321
2.5 Mean	1.022	1.5292	0.0466	0.0357	0.022	0.0292
5 cm	1.0373	1.5468	0.0675	0.0526	0.0373	0.0468
5 cm	1.0337	1.5346	0.0716	0.0297	0.0337	0.0346
5 cm	1.0307	1.5426	0.0611	0.0571	0.0307	0.0426
5 Mean	1.0339	1.5413	0.0667	0.0465	0.0339	0.0413
10 cm	1.0385	1.5734	0.0614	0.0855	0.0385	0.0734
10 cm	1.0874	1.5604	0.1266	0.0746	0.0874	0.0604
10 cm	1.0809	1.5663	0.1163	0.0785	0.0809	0.0663
10 Mean	1.0689	1.5667	0.1014	0.0795	0.0689	0.0667
**Total Mean**	**1.0416**	**1.5457**	**0.0716**	**0.0539**	**0.0416**	**0.0457**

**Table 2:** Distance measurement results when the box is a meter away in front of the robot or 45 degrees to the left or to the right.

Grid	Mean	Std. Dev.	Error
Front grid	1.0373	0.0614	0.0373
Front grid	1.0337	0.1266	0.0337
Front grid	1.0307	0.1163	0.0307
Front mean	1.0339	0.1014	0.0339
Right grid	1.0404	0.0443	0.0404
Right grid	1.0413	0.0469	0.0413
Right grid	1.0434	0.0468	0.0434
Right mean	1.0417	0.046	0.0417
Left grid	1.0224	0.0397	0.0224
Left grid	1.0271	0.0294	0.0271
Left grid	1.0255	0.0319	0.0255
Left mean	1.025	0.0337	0.025
**Total mean**	**1.0335**	**0.0604**	**0.0335**

## Supplementary Materials

The following are available at https://drive.google.com/open?id=1J4_SfymtGj-OKRigYzsapKRs1u8zDadx.
